# Medical Students’ Perceptions of the Doctor-Patient Relationship: A Cross-Sectional Study from Saudi Arabia

**DOI:** 10.7759/cureus.5053

**Published:** 2019-07-01

**Authors:** Ahmed M Fothan, Abdulaziz M Eshaq, Abdulrahman M Bakather

**Affiliations:** 1 General Surgery, Trauma & Orthopaedics, College of Medicine, Alfaisal University, Riyadh, SAU; 2 Internal Medicine, College of Medicine, Alfaisal University, Riyadh, SAU

**Keywords:** patient-doctor relationship, saudi arabia, patient-practitioner orientation scale, medical students

## Abstract

Introduction

The physician-patient relationship is at the heart of the art of medicine. Patient-centered care is rapidly evolving as the standard of care as well as the optimal vehicle to achieve high-quality healthcare and good clinical outcomes. This study aims to examine the attitudes of pre-clinical (third-year) students from Alfaisal University (Riyadh, Saudi Arabia) toward the physician-patient relationship.

Methods

This cross-sectional study took place during the spring 2017 academic year. All third-year students (n=210) were requested to voluntarily complete an online and anonymous questionnaire. The questionnaire covered students’ demographical characteristics (gender, nationality, and cumulative grade point average) and their response to a previously validated instrument, the Patient-Practitioner Orientation Scale (PPOS), which explores the attitudes of medical students toward the doctor-patient relationship.

Results

In total, 132 students participated in the survey (n=132/210) with an overall response rate of 62.9%. Most respondents were female (56.8%), non-Saudi citizens (53.0%), and high achievers (70.5%). Most of the mean scores on the PPOS statements indicated patient-centered attitudes - that is, mean scores were higher than “3”. The mean score for the overall PPOS was 4.0 ± 1.5, whereas the mean scores for the sharing and caring domains were 4.2 ± 1.5 and 3.8 ± 1.4, respectively. Univariate correlations between the students’ demographics and their mean scores for the sharing domain, caring domain, and overall PPOS showed no statistically significant differences (p > 0.05).

Conclusion

The pre-clinical medical students showed favorable patient-centered attitudes. There were no statistically significant differences between students’ demographics (gender, nationality, and academic performance) and PPOS scores (sharing domain, caring domain and overall score).

## Introduction

The art of medicine/clinical practice lies, to a greater extent, in the high-quality rapport between the physician and patient during clinical encounters. Nowadays, there is an increasing shift toward patient-centered care, in which “the physician tries to enter the patient’s world, to see the illness through the patient’s eyes ... so that the physician can understand each patient’s ideas, expectations, and feelings about the illness” [1].

Earlier reports have demonstrated that patient-centeredness and two-sided transparent communication fruitfully strengthen the physician-patient relationship. This, in turn, is favorably linked to improved healthcare, patient gratification, patient adherence to treatment, and eventually superior clinical outcomes [2-4].

Medical students are, largely, tomorrow’s workforce of 21st-century healthcare providers [5]. Thus, it is important to study the attitudes of medical students toward the physician-patient relationship. Appropriate students’ attitudes will be reinforced, whereas inappropriate ones can be rectified through curricular, extracurricular, and institutional (hospital-based) schemes during medical education. 

Several earlier studies from the United States, Nepal, Singapore and Pakistan have endeavored to scrutinize the attitudes of medical students toward the physician-patient relationship [1, 6-9]. However, to the best of our knowledge, no similar study was conducted in Saudi Arabia. Therefore, the aim of this study is to explore the attitudes of third-year medical students from Alfaisal University (Riyadh, Saudi Arabia) toward the physician-patient relationship. 

## Materials and methods

The study was conducted at the College of Medicine, Alfaisal University, Riyadh, Saudi Arabia. This cross-sectional study took place during the spring 2017 academic year. The study was approved by the Institutional Review Board (IRB) at Alfaisal University.

All third-year students (n=210) were requested to voluntarily complete an online and anonymous questionnaire. The reason for specifically choosing the third-year students was that they represent the most senior and mature pre-clerkship students, and their attitudes would be better thought out than that of their first- and second-year peers. The questionnaire covered students’ demographical data and their response to a previously validated instrument that explores the attitudes of medical students toward the doctor-patient relationship.

Students’ demographical data included gender, nationality, and cumulative grade point average (cGPA). Students’ attitudes toward the doctor-patient relationship were evaluated using the Patient-Practitioner Orientation Scale (PPOS) [7]. PPOS is a previously validated questionnaire and spans two primary domains: sharing and caring. The sharing domain explores the extent to which information is exchanged between the physician and the patient. On the other hand, the caring domain explores the extent of the trust, confidence, and warmth in the rapport between the physician and patient. Overall, there are 18 items in the questionnaire, divided equally between the sharing (n=9) and caring (n=9) domains. Statements 1, 4, 5, 8, 9, 10, 12, 15 and 18 deal with the sharing domain. Statements 2, 3, 6, 7, 11, 13, 14, 16 and 17 deal with the caring domain. Each item is evaluated on a six-point Likert scale statement, as follows: (1) strongly agree, (2) agree, (3) slightly agree, (4) slightly disagree, (5) disagree, and (6) strongly disagree. Higher scores (approaching a value of “6”) reflect more patient-centered attitudes, whereas lower scores (approaching a value of “1”) indicate more physician-centered attitudes.

The average Likert scale responses were presented as means ± standard deviations (SDs). Categorical data were presented as numbers and percentages. A two-tailed Mann-Whitney U-test was used to compare the average six-point Likert scale responses of male and female students. Statistical significance was determined as a p-value < 0.05.

## Results

In total, 132 students participated in the survey (n=132/210) with an overall response rate of 62.9%. Table 1 shows the students’ demographical data. All participants were third-year students (100%). The vast majority of students were females (56.8%), non-Saudi citizens (53.0%), and high-achievers (cGPA ≥ 3.00/4.00; 70.5%). 

**Table 1:** Demographics of all students (n=132)

Variable	n (%)
Academic year	
Third-year students	132 (100)
Gender	
Male	57 (43.2)
Female	75 (56.8)
Nationality	
Saudi	62 (47.0)
Non-Saudi	70 (53.0)
Cumulative grade point average	
≥3.00/4.00	93 (70.5)
<3.00/4.00	39 (29.5)

Table 2 shows the mean score of each PPOS statement based on the responses of all the students (n=132). The students' mean scores on the PPOS statements largely indicated patient-centered attitudes - that is, the mean scores are higher than “3”.

**Table 2:** Mean scores on the Patient-Practitioner Orientation Scale (PPOS) statements of all students (n=132)

#	Statement	Mean ± standard deviation
1	The doctor is the one who should decide what gets talked about during a visit	3.8 ± 1.6
2	Although healthcare is less personal these days, this is a small price to pay for medical advances	4.3 ± 1.7
3	The most important part of the standard medical visit is the physical exam	3.8 ± 1.7
4	It is often best for patients if they do not have a full explanation of their medical condition	4.9 ± 1.4
5	Patients should rely on their doctors’ knowledge and should not try to find out about their conditions on their own	4.3 ± 1.5
6	When doctors ask a lot of questions about a patient’s background, they are prying too much into personal matters	4.9 ± 1.4
7	If doctors are truly good at diagnosis and treatment, then the way they relate to patients is not that important	5.0 ± 1.4
8	Many patients continue asking questions even though they are not learning anything new	4.0 ± 1.4
9	Patients should be treated as if they were partners with the doctor, equal in power and status	3.0 ± 1.7
10	Patients generally want reassurance rather than information about their health	3.4 ± 1.5
11	If a doctor’s primary tools are being open and warm, the doctor will not have a lot of success	4.4 ± 1.3
12	When patients disagree with their doctor, this is a sign that the doctor does not have the patient’s respect and trust	4.1 ± 1.3
13	A treatment plan cannot succeed if it conflicts with a patient’s lifestyle or values	3.2 ± 1.5
14	Most patients want to get in and out of the doctor’s office as quickly as possible	3.3 ± 1.5
15	The patient must always be aware that the doctor is in charge	3.5 ± 1.4
16	It is not that important to know a patient’s culture and background in order to treat the person’s illness	4.9 ± 1.3
17	Humor is a major ingredient in the doctor’s treatment of the patient	3.6 ± 1.3
18	When patients look up medical information on their own, this usually confuses more than it helps	3.6 ± 1.4

Table 3 shows the mean scores of the sharing domain, caring domain, and overall PPOS for all students (n=132). The mean score for overall PPOS was 4.0 ± 1.5, whereas the mean scores for the sharing and caring domains were 4.2 ± 1.5 and 3.8 ± 1.4, respectively.

**Table 3:** Mean scores for the sharing subscale, the caring subscale and overall Patient-Practitioner Orientation Scale (PPOS) of all students (n=132)

Patient-Practitioner Orientation Scale (PPOS) Component	Mean ± standard deviation
Sharing subscale	4.2 ± 1.5
Caring subscale	3.8 ± 1.4
Overall Patient-Practitioner Orientation Scale (PPOS)	4.0 ± 1.5

Table 4 shows univariate correlations between the demographics (gender, nationality and cGPA) of all students (n=132) and their mean scores for the sharing domain, caring domain, and overall PPOS. No statistically significant differences were identified (p > 0.05).

**Table 4:** Correlation between the demographics of all students (n=132) and their mean scores for the sharing subscale, the caring subscale, and overall Patient-Practitioner Orientation Scale (PPOS)

	Sharing Subscale Mean ± standard deviation	Caring Subscale Mean ± standard deviation	Overall PPOS Mean ± standard deviation
Gender			
Male (n=57)	4.3 ± 1.5	3.8 ± 1.3	4.1 ± 1.4
Female (n=75)	4.2 ± 1.5	3.7 ± 1.5	3.9 ± 1.5
p-value	0.7050	0.6887	0.4364
Nationality			
Saudi (n=62)	4.2 ± 1.5	3.8 ± 1.4	4.0 ± 1.5
Non-Saudi (n=70)	4.3 ± 1.5	3.8 ± 1.4	4.0 ± 1.5
p-value	0.7050	1.0000	1.0000
Cumulative grade point average			
≥3.00/4.00 (n=93)	4.3 ± 1.5	3.8 ± 1.4	4.0 ± 1.5
<3.00/4.00 (n= 39)	4.1 ± 1.6	3.7 ± 1.4	3.9 ± 1.5
p-value	0.4944	0.7087	0.7273

## Discussion

This current study explored the attitudes of third-year medical students toward the physician-patient relationship using the PPOS, a previously validated questionnaire [7]. Overall, students demonstrated attitudes that favored patient-centeredness. To the best of our knowledge, this is the first cross-sectional study from Saudi Arabia that attempted to investigate the attitudes of pre-clinical medical students toward the patient-doctor relationship. It will be interesting to explore changes in attitudes when students transit from the pre-clinical to clinical (more intensive patient-doctor interactions) phases of medical education. Haidet and colleagues showed that students in their earlier years of medical school (pre-clinical years) were more likely to have patient-centered attitudes than those in the later years of medical school (clinical years) [6]. On the other hand, a study from Pakistan by Ahmad and partners found an opposite result, where students in later academic years (clinical years) were more likely to exhibit patient-centered attitudes [8].

Our study showed no statistically significant differences between students’ demographics and PPOS scores (p > 0.05). This might be, largely, attributable to the small sample size of the current study.

With respect to gender, two studies from the United States [7] and Nepal [9] showed that female gender was more likely associated with higher PPOS scores when compared to the male gender. Conversely, a study from Pakistan [8] identified no statistical difference across gender.

With respect to nationality, previous studies showed that students from foreign backgrounds (with regard to the country of medical training) [8] and European-American ethnicity [6] achieved higher PPOS scores. Saudi Arabia is, largely, a conservative society where the interactions between opposite genders (including patients and physicians) is limited. Although no statistically significant differences were identified between Saudi and non-Saudi national students, it will be interesting to explore Saudi patients’ likelihood to seek an open and mutual physician-patient relationship with Saudi/non-Saudi medical students during clinical encounters.

With respect to cGPA, no previous reports looked into a correlation between academic performance and students’ attitudes toward patient-centeredness. Although our results did not show a statistical difference, it is worth exploring this matter further in prospective studies. 

Medical school curricula should strive to instil more patient-centered attitudes among pre-clinical medical students, and this must also be continued throughout the clinical phases of medical education. A wide-ranging assortment of scholastic interventions should be put in place, such as curricular, extracurricular, and institutional (hospital-based) plans. The inputs of medical students are critically important and should be considered seriously during curricular development, as students are the primary stakeholders and tomorrow’s workforce of physicians. 

Our study has several limitations that ought to be mentioned. Such limitations include the small sample size, the limitation to a single medical school, and the self-reported study design that is subject to over- or under-estimation of perceived attitudes.

## Conclusions

The pre-clinical medical students showed patient-centered attitudes. There were no statistically significant differences identified between students’ demographics (gender, nationality, and academic performance) and PPOS scores (sharing domain, caring domain, and overall score).
